# Impact of genome architecture on the functional activation and repression of *Hox* regulatory landscapes

**DOI:** 10.1186/s12915-019-0677-x

**Published:** 2019-07-12

**Authors:** Eddie Rodríguez-Carballo, Lucille Lopez-Delisle, Nayuta Yakushiji-Kaminatsui, Asier Ullate-Agote, Denis Duboule

**Affiliations:** 1 Laboratory of Developmental Genomics, Department of Genetics and Evolution, University of Geneva, 1211 Geneva 4, Switzerland; 2 School of Life Sciences, Federal Institute of Technology, Lausanne, 1015 Lausanne, Switzerland; 3 Laboratory of Artificial and Natural Evolution, Department of Genetics and Evolution, University of Geneva, 1211 Geneva 4, Switzerland; 4 Collège de France, 75005 Paris, France; 5 Present Address: Laboratory for Developmental Genetics, RIKEN Center for Integrative Medical Sciences (IMS), 1-7-22 Suehiro-cho, Tsurumi-ku, Yokohoma, Kanagawa 230-0045 Japan

**Keywords:** Genome architecture, TAD, Polycomb, Tetrapod evolution, Enhancers, Regulatory landscapes

## Abstract

**Background:**

The spatial organization of the mammalian genome relies upon the formation of chromatin domains of various scales. At the level of gene regulation in *cis*, collections of enhancer sequences define large regulatory landscapes that usually match with the presence of topologically associating domains (TADs). These domains often contain ranges of enhancers displaying similar or related tissue specificity, suggesting that in some cases, such domains may act as coherent regulatory units, with a global on or off state. By using the *HoxD* gene cluster, which specifies the topology of the developing limbs via highly orchestrated regulation of gene expression, as a paradigm, we investigated how the arrangement of regulatory domains determines their activity and function.

**Results:**

Proximal and distal cells in the developing limb express different levels of *Hoxd* genes, regulated by flanking 3′ and 5′ TADs, respectively. We characterized the effect of large genomic rearrangements affecting these two TADs, including their fusion into a single chromatin domain. We show that, within a single hybrid TAD, the activation of both proximal and distal limb enhancers globally occurred as when both TADs are intact. However, the activity of the 3′ TAD in distal cells is generally increased in the fused TAD, when compared to wild type where it is silenced. Also, target gene activity in distal cells depends on whether or not these genes had previously responded to proximal enhancers, which determines the presence or absence of H3K27me3 marks. We also show that the polycomb repressive complex 2 is mainly recruited at the *Hox* gene cluster and can extend its coverage to far-*cis* regulatory sequences as long as confined to the neighboring TAD structure.

**Conclusions:**

We conclude that antagonistic limb proximal and distal enhancers can exert their specific effects when positioned into the same TAD and in the absence of their genuine target genes. We also conclude that removing these target genes reduced the coverage of a regulatory landscape by chromatin marks associated with silencing, which correlates with its prolonged activity in time.

**Electronic supplementary material:**

The online version of this article (10.1186/s12915-019-0677-x) contains supplementary material, which is available to authorized users.

## Background

Attempts to understand the spatial organization of the genome in the nucleus have recently led to models accounting for the relationship between genome structure and gene regulation (see [1]). The development of chromosome conformation capture techniques associated with deep sequencing has thus allowed the resolution of DNA interactions at a small scale [2]. These interactions can be either structural or functional, i.e., they can be present regardless of the transcriptional outcome or alternatively, they can fluctuate according to cell type-specific context depending upon the transcriptional status [3]. Constitutive contacts generally tend to fit the loop extrusion model, whereby the packed network of chromatin loops would form as a result of DNA extrusion by an ATP-dependent cohesin-based complex. In this model, the loops are stabilized whenever this cohesin ring meets two CTCF molecules bound with convergent orientations [4–6].

Chromatin is organized in several levels of interactions, loops, and domains. At the level of gene regulation, topologically associating domains (TADs) [7–9] usually match large domains of long-range gene regulation referred to as regulatory landscapes [10]. These structures are globally detected in all cell types and conserved across vertebrate species [7, 11–15]. The experimental depletion of either CTCF or cohesin subunits leads to a loss of both loop organization and TAD structure. Under these conditions, however, the effects upon gene transcription were limited and the formation of larger structures (compartments), which may also be functionally relevant, still occurred although in an altered manner [16–20].

Compartments contain chromatin domains labeled by various epigenetic marks. Inactive chromatin domains labeled by histone H3 lysine 27 trimethylation (H3K27me3), resulting from the presence of polycomb group protein complexes, have been associated either with compartment A [21] or with a compartment B1, distinct from the genuine heterochromatin B compartment [5], which segregate from other chromatin domains, possibly through phase separation [22, 23]. In addition, facultative heterochromatin (H3K27me3-positive) was shown to correlate with long-distance interactions either in stem cells [24–26] or during embryonic development [21, 27].

Distinct functional states associated with various chromatin structures are not as clear when TADs are considered. While several examples exist showing the functional coherence of multiple enhancer sequences present within one particular TAD [28–32], the definition of TADs as global independent regulatory units still lacks experimental evidence. In many instances, indeed, TADs include either series of enhancers with the same—or related—specificity or enhancers with distinct tissue-specific potentials but involved in the pleiotropic regulation of the same target gene(s). However, whether or not the entire TAD adopts a global on or off state, for example, related to a particular architecture, remains to be established.

A useful experimental paradigm to address this question is the mammalian *HoxD* gene cluster, a group of genes located at the intersection between two TADs displaying distinct functional specificities [33]. During limb development, enhancers in the telomeric TAD (3′-TAD) regulate the transcription of *Hoxd8* to *Hoxd11* in proximal limb bud cells. Subsequently, enhancers in the centromeric TAD (5′-TAD) control the *Hoxd9* to *Hoxd13* genes in distal limb bud cells [33]. These different sets of target genes responding to either one of the regulatory domains are determined by a robust boundary, centered around *Hoxd11* and relying upon a collection of bound CTCF sites. Genetic analyses in vivo revealed that this boundary was very resilient and that even a full deletion of the gene cluster was unable to merge both TADs into one single domain, likely due to a few remaining occupied CTCF sites [34].

The analysis of different developmental contexts where *Hoxd* genes are transcribed demonstrates that these two TADs are functionally exclusive from one another, i.e., the concomitant function of enhancers belonging to the two domains has not been observed thus far. This is due in part to the fact that the main target gene responding to 5′-TAD enhancers is *Hoxd13*, whose product, along with that of *Hoxa13*, has a negative effect over 3′-TAD enhancers through direct binding as observed in ChIP-seq experiments [32, 35]. These TAD-exclusive regulations can also be followed by the appearance of relevant chromatin marks: while the 3′-TAD is largely covered by H3K27ac marks in proximal limb bud cells, it becomes rapidly decorated by H3K27me3 marks at the time the 5′-TAD starts to be active in distal cells and to accumulate H3K27ac marks [33]. Therefore, in distal cells, H3K27me3 marks not only decorate the *Hoxd1* to *Hoxd8* genes (they are no longer transcribed) but also large DNA intervals within the 3′-TAD reflecting the off state of this regulatory landscape and re-enforcing the idea that it may behave as a coherent regulatory unit.

In this study, we challenged this hypothesis by investigating the effects of combining the two TADs into a single domain (a fused-TAD), after deletion of a large piece of DNA containing the *HoxD* cluster as well as other boundary elements. After merging, this fused-TAD contained enhancers that do not normally function in the same cellular context. We asked whether these various enhancers would keep their initial functional specificities or, alternatively, if they would all be active or repressed concomitantly, as a result of this new topological proximity. In addition, we used a set of engineered inversions, which disconnect the target *Hoxd* genes from their TADs to evaluate the functional and epigenetic behavior of these regulatory sequences in the absence of their target genes.

## Results

**Fig. 1:**
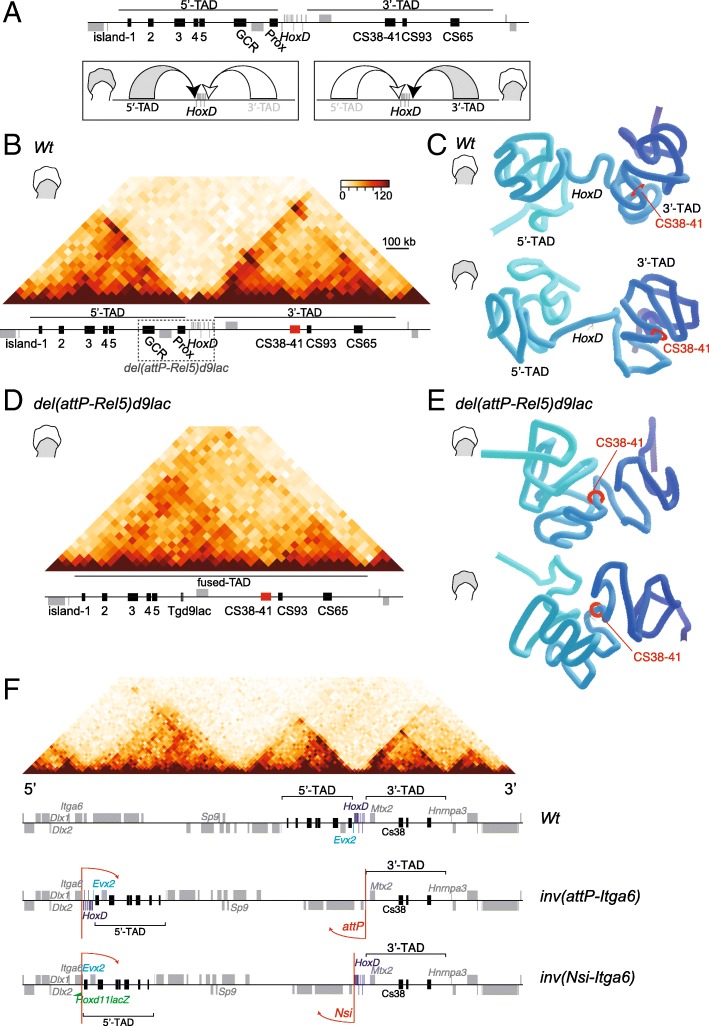
3D representation of the *HoxD* locus in control (*Wt*) and mutant limb buds. **a** Representation of the *HoxD* cluster and their associated regulatory domains (5′-TAD and 3′-TAD). The schemes below show the differential activation by the 5′-TAD and the 3′-TAD, in distal and proximal limb bud cells, respectively. **b** Hi-C map showing the distribution of TADs on either side of the *HoxD* locus in proximal limb and its associated genes (gray boxes) and regulatory regions (black and red boxes). The dashed rectangle illustrates the deletion in the *del(attP-Rel5)d9lac* allele. **c** Three-dimensional modeling of *HoxD*-associated TADs derived from Hi-C datasets obtained from wild-type (*Wt*) proximal (top) and distal (bottom) limb bud cells (schemes on the left). **d** Hi-C map showing the extent of the fused-TAD upon deletion of the *HoxD* locus in proximal limb cells, as well as the remaining genes (gray boxes) and regulatory regions (black and red boxes). **e** Comparative modeling from the *del(attP-Rel5)d9lac*-mutant proximal (top) and distal (bottom) limb bud cells. The red disk shows the position of region CS38-41 to be used as a reference point in all representations. The 3′-TAD and the 5′-TAD are indicated in **b** and **c**. The TADkit tool was used to model Hi-C datasets from [34]. **f** Schemes of two inverted mutant lines where the *HoxD* cluster is disconnected from its adjacent regulatory domains: the *inv(attP-Itga6)-* and *inv(Nsi-Itga6)*-mutant lines. On top, a Hi-C profile from limb cells with, below, the distribution of genes (gray) and regulatory regions (black) (chr2:71240000-76320000). The positions of both the 5′ and 3′-TADs are indicated with brackets. In the *inv(Nsi-Itga6)* allele, an inversion was generated between the *Itga6* and the *attP* breakpoints [37] separating the *HoxD* cluster from the 3′-TAD. In the *inv(Nsi-Itga6)* allele, the inversion occurred between the *Itga6* and the *Nsi* breakpoints [38], and hence, the *HoxD* cluster remains in contact with the 3′-TAD. In the latter inversion, a *Hoxd11lac* transgene (green flag) is inverted together with the DNA fragment

In order to better visualize the spatial distribution of the two TADs associated with the *HoxD* cluster (Fig. 1a, b), we modeled their structures in 3D by using Hi-C matrices [34] for both distal and proximal E12.5 limb bud cells (Fig. 1c) and the TADkit scripts package as a 3D modeling viewer [36]. In the wild-type condition, the *HoxD* cluster exerted a strong boundary effect and was thus positioned between the 3′-TAD and 5′-TAD regulatory domains, in both distal and proximal limb cells (Fig. 1c). In both tissues, a region called CS38-41 (Fig. 1, red disk) established a weaker boundary between two sub-TADs in the 3′-TAD. The structure and separation between the two regulatory domains were generally conserved between the two cell types, although with some minor differences.

We applied the same 3D modeling viewer to Hi-C datasets obtained with limb bud cells from the *HoxD*^*del(1-13)d9lac*^-mutant mouse stock (hereafter *del(1-13)d9lac*), which contains a deletion including the *HoxD* cluster [34] (see Additional file 1). In this mutant, the deleted DNA was substituted by a *Hoxd9lac* reporter transgene while the *Evx2* and *Lnpk* genes remained present. In the absence of the *HoxD* cluster, the 5′-TAD and 3′-TAD were still observed as independent structures despite a substantial shortening of the distance separating them (Additional file 1B-C). A clear spatial contraction was nevertheless scored between the 5′-TAD and the first sub-TAD in the 3′-TAD until region CS38-41 (Additional file 1B, C, red disk).

We next used the *HoxD*^*del(attP-Rel5)d9lac*^ (hereafter *del(attP-Rel5)d9lac*) Hi-C datasets from mutant limbs lacking ca. 350 kb of DNA including the *HoxD* cluster plus flanking regions (Fig. 1b, d, e). With this large deletion, the two TADs merged into one single structure (Fig. 1d, e) regardless of the cell type considered (distal or proximal limb bud), indicating that the TAD boundary had been entirely deleted. In this stock, the same *Hoxd9lac* transgene could be used as a transcriptional readout. The consolidation of both the 3′-TAD and the 5′-TAD into one single structure was obvious up to the CS38-41 region, whereas the most telomeric located sub-TAD in the 3′-TAD was somewhat less engulfed (Fig. 1e). We also performed the *C*-score analysis and attributed the interacting domains according to the positive *C*-score values to obtain the distribution of compartments A and B. We concluded that the position of the *HoxD* locus in compartment A, as well as the general compartment distribution along chromosome 2, was virtually identical between distal and proximal cells, when both the wild-type and the *del(attP-Rel5)d9lac* datasets were considered (Additional file 1D). Given that the topological changes in *del(attP-Rel5)d9lac* were limited to those domains adjacent to the cluster, we decided to investigate the impact on the activity of these regulatory domains upon the aforementioned deletion or using inversions where the *HoxD* cluster was disconnected either from the 3′-TAD or from the 5′-TAD (Fig. 1f).

### Transcription at the *HoxD* locus in the absence of the *HoxD* cluster

We assessed the transcription emanating from the *lacZ* reporter transgenes by whole-mount in situ hybridization (WISH) on E11.5 fetuses, using a *LacZ* RNA probe and could identify both the distal and proximal limb domains in the two *del(attP-Rel5)d9lac* and *del(1-13)d9lac* lines, though with subtle variations in their relative strengths (arrowheads Additional file 2A). Therefore, even in the complete absence of a TAD boundary (removed along with the deletion in the *del(attP-Rel5)d9lac* allele), the functional partition of proximal and distal enhancers occurred in a close-to-normal manner, with a clear separation between the two expression domains. While the distal domain overlapped well with the wild-type *HoxD* distal limb pattern (see *Hoxd10* and *Hoxd11* wild-type WISH for a comparison), the proximal domain was somewhat different in shape and position from the wild-type *HoxD* pattern (Additional file 2A), resembling the expression pattern of the *Hog* lncRNA [34] thus likely indicating some enhancer reallocation due to the novel topology of the locus.

**Fig. 2:**
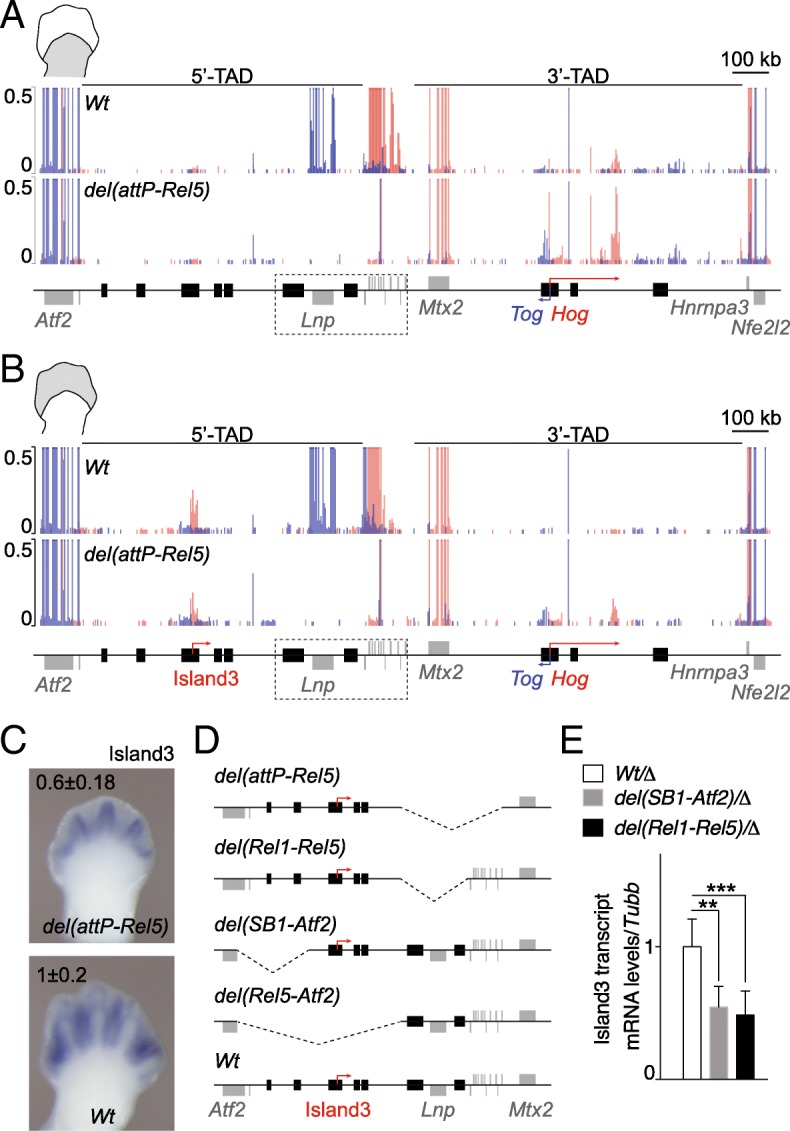
Transcript profiles at the *HoxD* locus in both control (*Wt*) and *del(attP-Rel5)d9lac*-mutant limb buds. **a**, **b** Normalized RNA-seq profiles of control (*Wt*) and mutant proximal (**a**) and distal (**b**) limb cells. Values from forward (red) and reverse (blue) strands are merged into the same graph. The positions of various genes and of Island3 are shown below. The dashed rectangles depict the *del(attP-Rel5)d9lac* deletion. The isolated signal around *Hoxd9* in the second tracks in **a** and **b** arises from the *Hoxd9/lacZ* reporter transgene present in the mutant line. The scale is set such that changes in non-coding regions can be better observed. *n* = 3. **c** WISH of Island3 eRNA in both *del(attP-Rel5)d9lac* and wild-type E12.5 forelimbs. qPCR values (mean ± SD) are shown on the top of each image. *n* = 6 for *Wt* and *n* = 4 for *del(attP-Rel5)d9lac*. **d** Schemes of the various deleted regions of the mutant lines used in **a** to **e**. **e** qPCR of Island3 eRNAs in E12.5 distal limb cells in two distinct partial deletions of the 5′-TAD. The mutant lines used were *del(SB1-Atf2)* (*n* = 4) and *del(Rel1-Rel5)* (*n* = 9), both balanced by the *del(Rel5-Atf2)* allele (where Island3 is deleted, abbreviated by Δ in the legend). Results were compared to *del(Rel5-Atf2)/+* samples as controls (white bar, *n*=12). Bars show mean ± SD. Welch’s *t* test **p = 0.0026 and ***p < 0.0001

In order to have a more precise account of these local modifications in transcriptional responses following the fusion of the two TADs, we carried out RNA-seq for both proximal and distal cell populations in both control (*Wt*) and *del(attP-Rel5)d9lac*-mutant limbs at E12.5. In control proximal cells, transcripts were expectedly detected for *Hoxd* genes, for the flanking *Lnpk* and *Mtx2* genes as well as for the *Hog* and *Tog* lncRNAs, two non-coding RNAs localized within the 3′-TAD and normally responding to enhancers located in this domain [34, 39] (Fig. 2a, top). In control distal cells, while the expression of the latter two lncRNAs was undetectable, digit-specific transcripts were scored over the Island3 region both by RNA-seq and by WISH (Fig. 2b, top; Additional file 2B), a region previously defined as a distal cell-specific enhancer [40]. Therefore, we used these non-coding RNAs (*Hog*, *Tog*, and Island3) as proxies to evaluate the activity of their surrounding proximal versus distal enhancers in the two deletion alleles that removed all target *Hoxd* genes.

In proximal *del(attP-Rel5)d9lac*-mutant limb cells, the *Hog* and *Tog* RNA levels substantially increased (adjusted *p* value from DESeq2 analysis of 1.75e−10 and 6.72e−22, respectively) while at the same time, the mRNA levels corresponding to the *Mtx2* and *Atf2* housekeeping genes remained approximately the same (adjusted *p* value = 1.00) (Fig. 2a, bottom; Additional file 2C). Transcripts for *Hoxd* genes and *Lnpk* had expectedly disappeared after the deletion, yet a signal remained for *Hoxd9* reflecting the transcription of the reporter gene left in place. Of note, the level of Island3 eRNA did not seem to increase in the deleted configuration. Therefore, while in the absence of target *Hoxd* genes, proximal enhancers within the former 3′-TAD were partly re-allocated towards the neighboring *Hog* and *Tog* promoters, they did not seem to affect Island3 expression, despite the removal of the TAD boundary (Fig. 2a, bottom; Additional file 2C).

In distal *del(attP-Rel5)d9lac*-mutant limb cells, the level of Island3 eRNA decreased in the deleted configuration (Fig. 2b, c). While this transcript did not appear as differentially expressed in our RNA-seq whole genome analysis due to restrictive parameters (46% reduction mutant versus control, adjusted *p* value = 1.4e−4; Additional file 2D), it showed a significant reduction by qPCR (40% reduction mutant versus control, Welch’s *t* test *p* value = 0.0166) and by WISH (Fig. 2c). To assess whether this reduction was due to the observed slight shrinking in the size of the presumptive digit domain in mutant limbs, we selected the ten genes with higher log2 fold change in RNA normalized read counts in distal versus proximal control limb cells. We next compared the normalized read counts of these mRNAs between mutant and wild-type distal limbs. Although some of them showed lower normalized read counts in *del(attP-Rel5)d9lac*, only *Dbx2* and the *1810046K07Rik* RNA appeared significantly reduced in the DESeq2 comparison (70% decrease, adjusted *p* value = 5.7e-28, and 82% decrease, adjusted *p* value = 5.3e−3, respectively, Additional file 2E). Therefore, it is likely that the decrease in Island3 transcripts was due to the deletion of both the GCR and Prox distal enhancers, as also suggested by the deletion of the Rel1 to Rel5 region (Fig. 2d, e).

A comparable outcome was observed as well in the deletion from SB1 to *Atf2*, which removes two different enhancers (islands 1 and 2) on the other end of the regulatory domain (Fig. 2d, e). Noteworthy, neither of the housekeeping transcription units was transcribed more efficiently. However, a significant increase in *Hog* and *Tog* lncRNAs was scored, while these two genes are normally silent in distal cells where the 3′-TAD has been switched off (Fig. 2b, Additional file 2D). Such an upregulation could illustrate either a weakening in the repression of the 3′-TAD in distal cells or novel interactions between distal enhancers located in former 5′-TAD and the two lncRNAs’ loci, following the deletion of the TAD boundary.

Altogether, these results indicate that distal and proximal limb enhancers that are normally located in different TADs and which work in an antagonistic manner can achieve close-to-proper functional specificities when they are regrouped into a single fused TAD.

### Changes in chromatin marks after TADs fusion

**Fig. 3:**
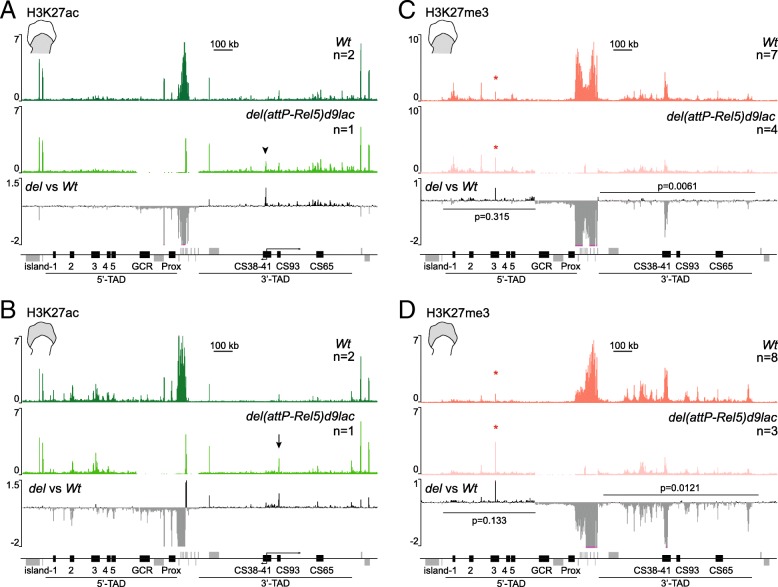
Distribution of H3K27ac and H3K27me3 marks over the *HoxD* cluster and its flanking TADs in both control (*Wt*) and *del(attP-Rel5)d9lac* proximal and distal limb bud cells. **a**, **b** H3K27ac ChIP profiles from proximal (**a**) and distal (**b**) limb cells. Control is on top, and the *del(attP-Rel5)d9lac* profile is shown below along with the difference of deleted versus control ChIP data (*del* versus *Wt*). The arrowhead in **a** depicts the shared *Hog* and *Tog* start site (see also the divergent arrows below). The arrow in **b** indicates the CS93 enhancer. **c**, **d** H3K27me3 ChIP profiles from proximal (**c**) and distal (**d**) limb bud cells. Control is on top, and the *del(attP-Rel5)d9lac* track is shown below along with a comparison profile showing the difference between mutant and wild-type profiles. The data were averaged between different experiments (*n* is shown on the right). The red asterisks indicate artifactual peaks. The signal from the *Hoxd9* region in the deleted allele corresponds to the *Hoxd9/lacZ* transgene

To confirm these observations, we looked at the acetylation of H3K27, a histone modification that positively correlates with transcriptional activity, and compared proximal and distal E12.5 limb bud cells derived from both control and *del(attP-Rel5)d9lac*-mutant fetuses. In proximal cells, the distribution of H3K27ac marks in the mutant material was as in control (wild-type) cells (Fig. 3a). H3K27ac modifications were found enriched in the active 3′-TAD while depleted from the inactive 5′-TAD. The amount of H3K27ac was slightly increased over a large region in the 3′-TAD in mutant cells, with a particular increase at the transcription start site of both *Hog* and *Tog* (Fig. 3a, 120% increase, arrowhead) thus confirming the previously described increased in RNA levels (Fig. 2). The distribution of H3K27ac marks over the 5′-TAD was comparable in control and mutant proximal cells (Fig. 3a, see *del* versus *Wt*).

In *del(attP-Rel5)d9lac*-mutant distal cells, an increase in H3K27ac was scored at region CS38-41 (Fig. 3b, 75% increase), which correlated with the activation of these two lncRNAs in these mutant cells, while they are normally silent in their wild-type counterparts (Fig. 2b). In addition, a strong increase in this histone H3 mark was scored in CS93 (Fig. 3b, arrow, 75% increase), a region characterized as a proximal limb enhancer [15]. The general distribution of H3K27ac appeared slightly increased throughout the 3′-TAD in mutant cells when compared to control (Fig. 3b). This slight increase in the 3′-TAD activity was also noticeable when analyzing proximal mutant tissue. A striking effect was however observed in H3K27ac coverage over the 5′-TAD in mutant versus control distal cells. A substantial loss of H3K27ac was indeed scored over the regulatory regions island1, 2, 4, and 5 (Fig. 3b, about 40% decrease). This effect was not as evident over island3, i.e., in the region where the enhancer transcript was detected in both control and mutant distal cells (Fig. 2b). Therefore, in distal cells, the fusion of the two TADs and removal of target genes seemed to weaken the transcriptional activity in the 5′-TAD while maintaining the activity of the 3′-TAD well above the silencing observed in control distal cells.

To back up this observation, we looked at the distribution of H3K27me3 marks, which are associated with gene silencing. In control proximal limb cells, H3K27me3 were detected over the 3′-TAD at E12.5 (Fig. 3c), i.e., when this landscape is still functionally active, due to the presence of a large percentage of negative cells in the dissected material (see [33]). In distal cells, where the 3′-TAD is switched off, a robust increase was detected with a strong coverage of the entire domain (Fig. 3d). In proximal cells, H3K27me3 marks were also scored over the silent 5′-TAD regulatory islands, a labeling that mostly disappeared upon the activation of these regulatory islands in distal cells (Fig. 3c).

The H3K27me3 profiles obtained with the *del(attP-Rel5)d9lac*-mutant limb buds were in agreement with the distributions of both the H3K27ac marks and the transcripts. In proximal mutant cells, the profile was globally similar to that seen in control cells showing no salient difference along the remaining part of the former 5′-TAD (6% increase, *p* value = 0.32), whereas a 22% decrease was scored over the remnant of the 3′-TAD (*p* value = 0.0061). This decrease was mostly centered on region CS38-41, which showed a 51% decrease in the mutant (Fig. 3c). In distal cells, the same effect was scored, yet with a higher magnitude. H3K27me3 marks were indeed heavily depleted from the 3′-TAD (50% reduction, *p* value = 0.012) whereas they were found mildly yet not significantly enriched all over the remaining 5′-TAD region containing the regulatory islands (16% increase, *p* value = 0.13) (Fig. 3d). Therefore, these results confirmed that in mutant cells carrying the combined fused-TAD, the former 3′-TAD landscape is globally overactive in distal cells at the expense of 5′-TAD enhancers, which appear less active than in their native context.

### Recruitment of PRC complexes at the *HoxD* cluster

**Fig. 4:**
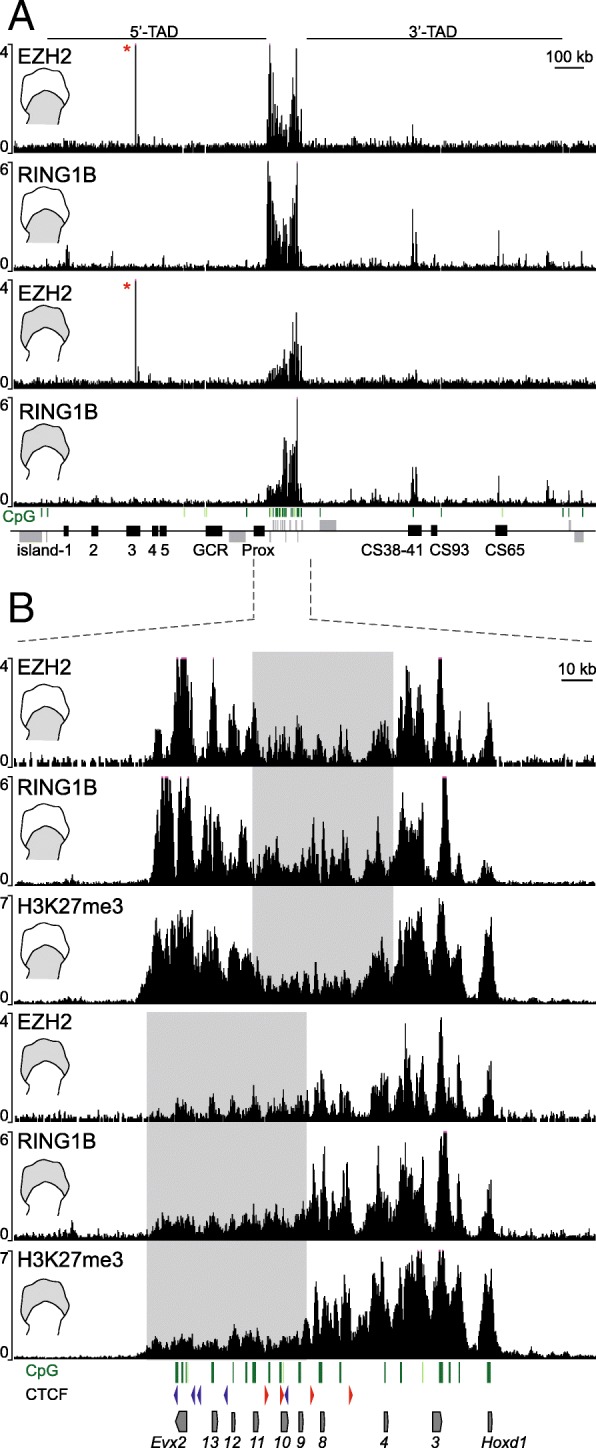
Distribution of PRC1 (RING1B) and PRC2 (EZH2) over the *HoxD* cluster and regulatory landscapes in limb bud cells. **a** EZH2 and RING1B ChIP profiles in proximal (top two panels) and distal (bottom two panels) E12.5 limb bud cells. The CpG distribution is shown as green bars on top of the gene diagram. The red asterisk indicates an artifactual signal. **b** Magnification of the *HoxD* cluster showing the distribution of EZH2 and RING1B in proximal and distal limb cells. H3K27me3 ChIP tracks are shown for each tissue. The CpG islands are shown as green bars, and the CTCF and their orientations are depicted as blue (reverse strand) or red (forward strand) arrowheads

Polycomb repressive complexes (PRC1 and PRC2) are generally associated with lack of gene expression and usually recruited to the CpG islands close to the transcriptionally active regions [24, 41, 42]. In this context, the massive presence of H3K27me3 marks over the 3′-TAD, a region largely devoid of coding units, raised the question of its recruitment. We looked at the presence of both EZH2 and RING1B, two components of PRC2 and PRC1, respectively. ChIP-seq experiments revealed that EZH2 was located mostly within the *HoxD* cluster (Fig. 4a). Outside the gene cluster, a weak enrichment was scored over region CS38-41 in proximal cells, which appeared even weaker in distal cells. Altogether, the two gene deserts were generally devoid of PRC2. A comparable conclusion was reached regarding the prevalence of signal at the cluster, with the analysis of the PRC1 component RING1B, even though some enrichment was detected on the gene deserts, generally over the 3′-TAD and particularly over the CS38-41 and CS65 regions, without any striking difference between distal and proximal cells (Fig. 4a). Some light differences were scored in the 5′-TAD, where a few regulatory regions appeared specifically decorated in proximal tissue but devoid of RING1B in distal tissue (compare Island1 and Island4 in Fig. 4a).

Within the *HoxD* cluster itself, the distribution of both EZH2 and RING1B nicely matched the coverage by H3K27me3 marks (Fig. 4b) [32, 33]. In proximal cells, the coverage was minimal over those genes active in response to the 3′-TAD enhancers (from *Hoxd8* to *Hoxd11*, rectangle in Fig. 4b, tracks 1 and 2) while in distal cells, genes responding to 5′-TAD enhancers were bound only weakly by either PRC2 or PRC1 (from *Hoxd13* to *Hoxd9*, Fig. 4b, rectangle in tracks 4 and 5). The EZH2 signals were significantly enriched at the CpG islands and over the coding regions, whereas the distribution of PRC1 was broader (Fig. 4b), suggesting a recruitment of PRC2 by CpG islands [24, 42, 43, 41].

Considering that H3K27me3 covered both *Hox* genes and their regulatory landscapes whereas PRC complexes were mostly recruited to the *HoxD* cluster itself, we wondered whether the reduction of H3K27me3 marks along the 3′-TAD in *del(attP-Rel5)d9lac*-mutant proximal cells could result from the mere absence of the *HoxD* gene cluster. To this aim, we used the engineered *HoxD*^*inv(attP-Itga6)*^ inversion (hereafter *inv(attP-Itga6)* where the *HoxD* cluster was disconnected from the 3′-TAD and displaced circa 3 Mb away while preserving both its integrity and its association with the 5′-TAD [37] (Fig. 1f).

**Fig. 5:**
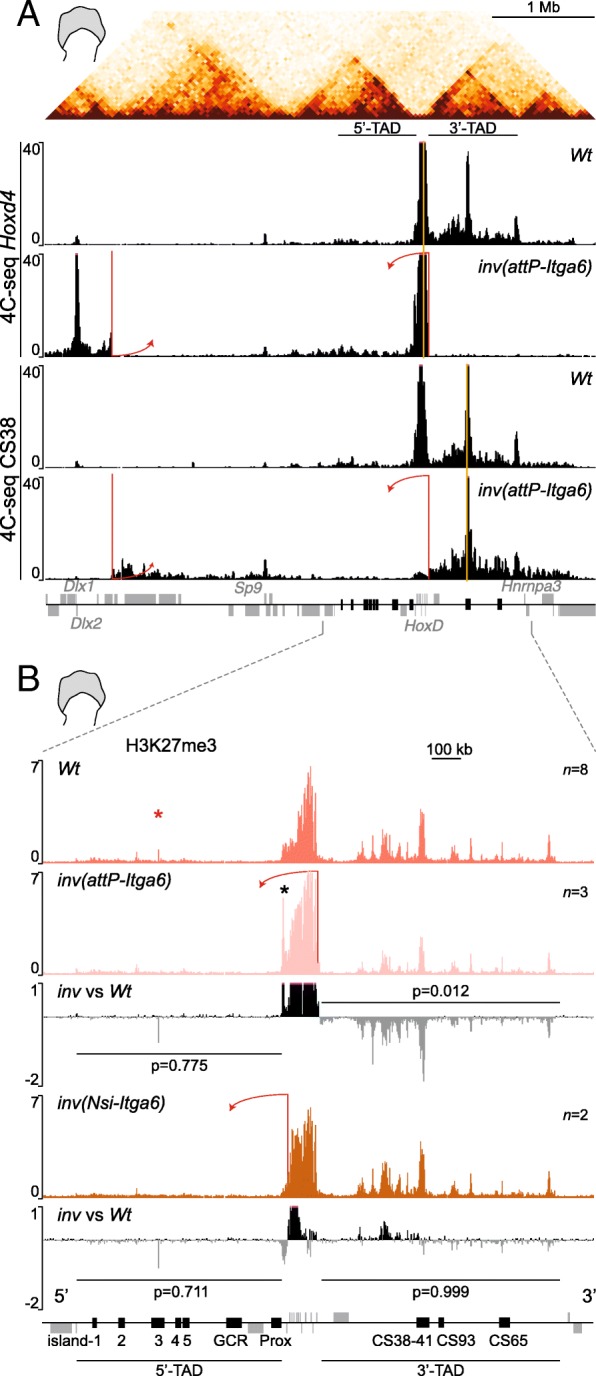
Epigenetic changes after disconnecting the *HoxD* cluster from its flanking 3′-TAD. **a** On top, a Hi-C profile of distal limb bud cells shows the *HoxD*-associated TADs. The panels below show a comparison of 4C-seq tracks between control (*Wt* from [34]) and *inv(attP-Itga6)*-mutant distal limb cells. Either the *Hoxd4* gene (top two panels) or the CS38 region (bottom two panels) was used as baits (yellow vertical bars). The red bars indicate the locations of the loxP sequences used to generate the inversion. After inversion, contacts between *Hoxd4* and the 3′-TAD are all lost, while they barely change when region CS38 is used as bait. **b** H3K27me3 ChIP profiles in control (*Wt*) and either the *inv(attP-Itga6)* inversion (top two profiles) or the *inv(Nsi-Itga6)* inversion (bottom two profiles). Below each mutant track, a comparison between mutant and control data is shown. The red bars indicate the inversion breakpoints. In the *inv(attP-Itga6)* track, an additional peak appears at the 5′ extreme of the *HoxD* cluster (black asterisk), corresponding to an ectopic sequence introduced when building the attP breakpoint*.* The red asterisk indicates an artifactual signal. The number of replicates is shown for each track. Below each mutant track, a difference profile of mutant versus control signals is represented

We verified that the genomic interactions between *Hoxd* genes and the 3′-TAD were abrogated in this *inv(attP-Itga6)* inverted allele by performing a 4C-seq analysis in mutant and control distal limb cells, with *Hoxd4* and CS38 as viewpoints (Fig. 5a). Expectedly, the contacts established by *Hoxd4* were no longer oriented towards the 3′-TAD in the inversion allele, when compared with control (Fig. 5a, tracks 1 and 2). In this inverted allele, interactions were now established de novo between *Hoxd4* and a region around the *Itga6* and *Dlx1/Dlx2* genes, near the inversion breakpoint. Also, contacts with the 5′-TAD were slightly increased. Furthermore, when region CS38 was used as a viewpoint, interactions with the *HoxD* cluster were largely lost and most contacts remained within the 3′-TAD itself (Fig. 5a, tracks 3 and 4).

In this inverted configuration, the global amount of H3K27me3 marks deposited over the 3′-TAD was substantially lower when compared to the control cells (Fig. 5b, tracks 1 and 2; Additional file 3). This decrease was not observed when another inversion was used as a control. In the *HoxD*^*inv(Nsi-Itga6)*^ allele (hereafter *inv(Nsi-Itga6)* [38], the *HoxD* cluster remains in place yet the 5′-TAD is inverted towards the same *Itga6* breakpoint (Fig. 1f). Therefore, these two inversions are identical except that one contains the *HoxD* cluster whereas the other does not (Fig. 5b, arrows in tracks 2 and 4; Additional file 3). In the *inv(Nsi-Itga6)* inversion allele, the enrichment of H3K27me3 over the 3′-TAD remained unchanged either in distal cells (*p* value = 0.999), or in proximal cells (*p* value = 0.50), as was the case for the *inv(attP-Itga6)* allele (Fig. 5b and Additional file 3). Altogether, these results and those obtained with the *del(attP-Rel5)d9lac* allele suggest that the presence of *Hoxd* genes was necessary to achieve a full spread of H3K27me3 marks over the 3′-TAD, up to 800 kb in far*-cis*.

Of note, this effect was restricted to the 3′-TAD, a conclusion reached after zooming out and looking at a 10-Mb interval surrounding the *HoxD* cluster. In control distal cells, the distribution of H3K27me3 marks was enriched selectively over the 3′-TAD, terminating abruptly at its TAD boundary with no further telomeric spreading. In mutant *del(attP-Rel5)d9lac* distal cells, despite the large reduction of H3K27me3 signals, the remaining coverage was also restricted up to the new telomeric boundary of the fused-TAD (Additional file 4A, B) without extending into neighboring TADs (R1 and R4 in Additional file 4A, 4B). Similar results were obtained when comparing the mutant *inv(attP-Itga6)*. In all cases, though to a different extent, the TAD structure appeared to determine the extent of H3K27me3 spreading.

### H3K27me3 inheritance and clearance

**Fig. 6:**
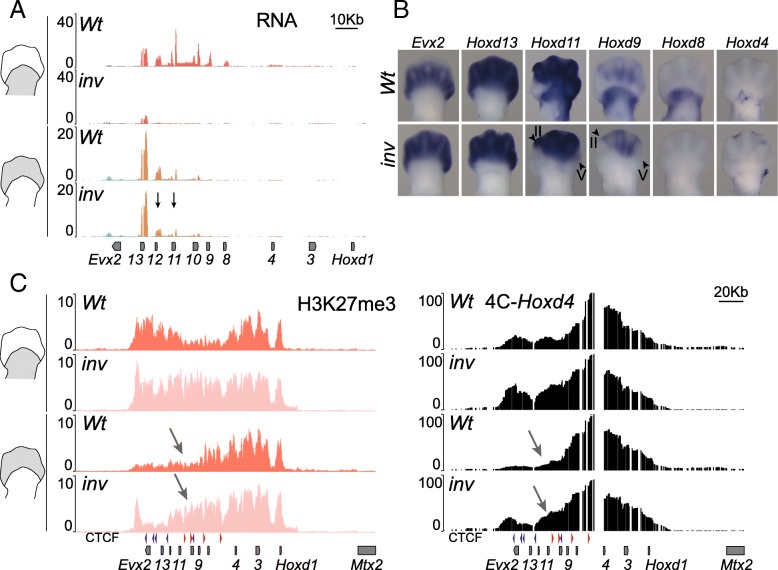
*Hoxd* gene expression in the limbs in the absence of the 3′-TAD. **a** Normalized RNA-seq profiles of control (*Wt*) and *inv(attP-Itga6)*-mutant proximal (**a**) or distal (**b**) limb bud cells. Black arrows indicate the decreased RNA quantity over *Hoxd12* and *Hoxd11* in distal tissue (bottom two tracks) while expression has almost fully disappeared in proximal limb cells (top two tracks). **b** WISH of *Hoxd4*, *Hoxd8*, *Hoxd9*, *Hoxd11*, *Hoxd13*, and *Evx2* in E12.5 forelimb buds. The arrowheads indicate digits II and V. **c** On the left, comparison of H3K27me3 signal over the *HoxD* cluster in either proximal (top two tracks) or distal (bottom two tracks) between control (*Wt*) and mutant *inv(attP-Itga6)* specimen. The CTCF sites are shown below. The arrows point to the extension of the H3K27me3 negative domain over the *Hoxd11* region in mutant *inv(attP-Itga6)* distal cells (fourth track), when compared to control cells (third track). On the right, 4C-seq tracks showing interactions inside the *HoxD* cluster when *Hoxd4* is used as bait (*Wt*: data from [34]). The arrows indicate a robust gain of interaction over *Hoxd11* to *Hoxd12* region in *inv(attP-Itga6)*-mutant distal cells

In the *inv(attP-Itga6)* allele, the 3′-TAD proximal enhancers were disconnected from their target *Hoxd3 to Hoxd11* genes, similar to a previous case when a deletion of 3′-TAD was used [33]. In both cases, the expression of these target genes was expectedly lost in proximal cells of the forelimb buds (Fig. 6a, b; see also [33]). Unexpectedly, however, both the quantity and distribution of *Hoxd9* and *Hoxd11* mRNAs were also reduced in distal cells (see digits II and V), where these genes are under the control of 5′-TAD enhancers (Fig. 6a, b, arrows and arrowheads, respectively). This surprising observation was explained by the lineage transmission, from proximal to distal cells, of H3K27me3 marks abnormally present in *Hoxd* genes in the absence of 3′-TAD [33].

To further substantiate this possibility, we analyzed the precise distribution of H3K27me3 marks over the *HoxD* cluster in the *inv(attP-Itga6)* allele. In proximal cells, we found a high and homogeneous coverage of this histone modification, from *Hoxd1* up to *Evx2*, unlike in the control allele where the DNA interval between *Hoxd8* and *Hoxd11* was transcriptionally active and hence depleted from this mark (Fig. 6c, tracks 1 and 2). This homogeneous distribution of H3K27me3 over the gene cluster in the mutant allele reflected the complete lack of *Hoxd* expression in proximal cells (Fig. 6a, tracks 1 and 2; Fig. 6b). In control distal cells, the region from *Evx2* to *Hoxd9* was depleted of H3K27me3 marks, as expected from the active regulation of the 5′-TAD enhancers.

In inverted mutant distal cells, however, an abnormally high H3K27me3 coverage was scored over the *Hoxd9* to *Hoxd11* region (Fig. 6c, arrow in track 4), which corresponded to the decrease in transcript levels observed for these genes in these cells (Fig. 6a, tracks 3 and 4). This increase in H3K27me3 was not observed in the *inv(Nsi-Itga6)*, where these genes are normally expressed in proximal tissue (Additional file 5). Because distal limb bud cells are the descendants in the lineage of proximal cells (see [44]), we explain this negative effect over the 5′-TAD regulation by the transmission of H3K27me3 marks from proximal to distal cells. These marks were ectopically detected over the *Hoxd4* to *Hoxd11* region in proximal cells due to the lack of contacts between proximal enhancers and their target *Hoxd* genes thus preventing their transcriptional activation. Of note, *Hoxd13* and *Evx2* transcript levels remained unchanged in the mutant allele when compared to control.

We assessed whether this ectopic gain of H3K27me3 in proximal cells would translate into a change in the extent of the negative chromatin sub-domain formed at *Hox* loci by H3K27me3-enriched sequences [45, 46]. We carried out 4C-seq by using *Hoxd4* as a viewpoint and noticed that in proximal cells, contacts established by *Hoxd4* clearly extended over the 5′ part of the cluster in the mutant allele, in agreement with the gain of H3K27me3. These contacts were also observed, though to a slightly lesser extent, in mutant distal cells, again correlating with the persistence of H3K27me3 marks (Fig. 6c, arrow in track 4).

## Discussion

During limb development, the two TADs associated with the *HoxD* cluster are either transcriptionally active or repressed, in an exclusive manner. Initially, the 3′-TAD enhancers are active and control the first wave of *Hoxd* transcription in early limb buds. Subsequently, these enhancers activate *Hoxd* genes in proximal structures such as the forearms [33]. In the second phase, the 5′-TAD enhancers become activated in distal limb cells (the future hands and feet) while the 3′-TAD concomitantly terminates operating and becomes covered by negative H3K27me3 marks [33, 40]. This bimodal regulation in TAD activities is necessary to organize each of the proximal and distal *Hox* expression domains, which are essential for proper limb development [47–50].

### A fused-TAD

Previous studies of this functional switch between these two TADs have suggested that they could represent coherent and independent regulatory units, i.e., that the 3D structure itself may participate in the global functional output of the system. In this view, a TAD could be either functionally permissive or refractory to the implementation of all the enhancers it may contain [32] thus representing an additional regulatory layer. In the case of both 3′-TAD and 5′-TAD, only one of them is licensed to work at a time since the presence of HOX13 proteins, partly determined through the activation of the 5′-TAD, leads to the repression of the 3′-TAD [32]. We wondered how this functional exclusivity would translate after the fusion of the two structures, in a situation where both proximal and distal enhancers would be included in the same fused-TAD. In this fused-TAD indeed, several enhancers normally present in the 5′-TAD, i.e., with a distal specificity, are now located along with the enhancers normally displaying a proximal specificity due to their location within the 3′-TAD. Since their target *Hoxd* genes were absent, we assessed their functionality by using three transcription units as readouts: an eRNA encoded by Island3 within former 5′-TAD, the *Hog* and *Tog* lncRNAs encoded within former 3′-TAD and a *Hoxd9/lacZ* reporter transgene positioned exactly between the former two TADs.

The analysis of *lacZ* mRNA revealed the presence of distinct proximal and distal expression domains, suggesting that the presence of the two kinds of enhancers in the same fused-TAD did not drastically affect neither their global functional specificities nor their mode and sequence of action. However, the proximal domain was distinct from what is normally observed in the wild-type limbs, despite the remaining presence of all known proximal enhancers in the two deleted alleles. In fact, it resembled in its position and shape to the expression domain of the lncRNA *Hog*, which is located within the 3′-TAD surrounded by proximal enhancers. In this case, the absence of target genes and their associated CTCF sites may have led to reallocations in enhancer-promoter contacts, as also suggested by the upregulation of *Hog* and *Tog* lncRNAs in proximal mutant cells. Therefore, the final transcription readout of 3′-TAD enhancers may slightly vary in space and time depending on how the target promoters are organized and on their local topology.

In addition, *Hog* and *Tog* transcripts were scored in mutant distal cells, while completely switched off in control distal cells. We interpret this as a response of these lncRNAs to the remaining 5′-TAD enhancers, in the absence of the TAD boundary. Also, the global repression of the 3′-TAD in mutant distal cells was not implemented as efficiently as in control cells, thus contributing to this light upregulation. At this point, it is difficult to associate the upregulation of these lncRNAs either with the lack of 3′-TAD repression or to de novo established promoter-enhancer interactions, given that both events would have a similar transcription outcome. However, when comparing to other remaining genes, these effects were specific for *Hog* and *Tog*, and hence, it seems that they may engage with active enhancers. Housekeeping genes located within or in the vicinity of the former 3′-TAD, such as *Mtx2*, *Hnrnap3*, or *Atf2*, were transcriptionally unaffected after fusion of the TADs, as these genes generally escape the regulations exerted by global enhancers in these landscapes.

In parallel with the maintenance of proximal enhancer activity in distal cells of the fused-TAD, the level of Island3 eRNAs was slightly reduced. While this RNA was exclusively present in distal cells, the same regulatory region showed a diminution of its transcriptional activity after merging of the two TADs, as if the fused-TAD was globally pushed towards a proximal type of regulation. A clear distal domain was nevertheless still detected with the *lacZ* expression pattern, demonstrating the activity of at least some distal limb enhancers and suggesting that the reduction in Island3 eRNA level may also be caused by the deletion of some distal enhancers in the fused-TAD.

This tendency of the fused-TAD to adopt a type of regulation globally more proximal than distal was reinforced by the analyses of chromatin marks. In distal cells, the fusion between the two TADs was indeed accompanied by a decrease in H3K27ac coverage in several enhancers located in the former 5′-TAD. In contrast, H3K27ac marks in mutant distal cells were more abundant in the former 3′-TAD region, i.e., over proximal enhancers, than in control distal cells where these marks rapidly disappear [33]. In general terms, however, H3K27ac deposition associated with enhancer activation in mutant cells was still observed as in control cells, indicating that the former 3′-TAD enhancers were still active in proximal limb bud cells and the former 5′-TAD enhancers in distal cells. The difference was observed in the balance between these two types of regulations rather than in their implementation.

The profile of H3K27me3 marks confirmed these observations. In distal cells where the 3′-TAD is normally inactive, the amount of H3K27me3 was significantly reduced in mutant versus control cells, as if the “proximal regulation” had not been entirely switched off in distal cells. In parallel with both the decrease of Island3 eRNAs level and the decrease in H3K27ac, the distribution of H3K27me3 marks appeared increased in the former 5′-TAD. Altogether, these results suggest that when mixed into a single fused-TAD, the proximal regulation tends to take the lead over the distal regulation, with proximal enhancers being active for too long, even in distal cells where distal limb enhancers seem to be somewhat under-active. A potential mechanism may involve the reported effect of HOX13 proteins in the termination of 3′-TAD regulation, combined with the novel chromatin architecture of the fused-TAD. In the absence of HOXD13 proteins, deleted from the fused-TAD, the dose of HOXA13 should be sufficient to secure the repression of 3′-TAD and thus to implement the switch in regulations [32]. However, the new chromatin configuration of this part of 3′-TAD when included in the fused-TAD may affect the negative function of HOXA13, leading to a partial inhibition only and hence to an improper switch off of proximal enhancers.

### TAD-specific and long-range effect of PRC silencing

Our results also provide some indications as to how PRC silencing propagates *in-cis* at a distance (see [51, 52]). Within the *HoxD* cluster itself, we show that PRC2 recruitment selectively occurs at the CpG islands, as previously proposed (e.g., [24, 52]). In addition, however, H3K27me3 marks were found throughout the 3′-TAD (over ca. 800 kb) in distal cells, where proximal enhancers have terminated their function, even though H3K27me3 marks were shown not to spread outside the *HoxD* cluster in a linear manner [53]. In the *del(attP-Rel5)d9lac* deletion, in the almost complete absence of CpG islands in and around the *HoxD* cluster, the enrichment by H3K27me3 marks in the 3′-TAD was severely reduced in distal cells, indicating that indeed the recruitment of PRC2 complexes over the *HoxD* cluster was mandatory to start covering the telomeric regulatory landscape by H3K27me3 marks, concomitantly to its functional inactivation. Some H3K27me3 coverage was nevertheless detected in the 5′-TAD and more substantially in the 3′-TAD, perhaps due to the presence of both the *Hoxd9/lacZ* reporter transgene and the *Hog* and *Tog* transcription start sites.

The coverage by H3K27me3 marks in control distal cells outside the *HoxD* cluster itself, i.e., in a region that is not particularly enriched in PRC2, exactly matched the extent of the 3′-TAD containing those *Hoxd* genes inactivated in distal cells and hence heavily covered by PRC2, PRC1, and H3K27me3. Such an effect was not scored in any other region in the 10 Mb surrounding the *HoxD* locus. This result suggests that the global inactivation of the 3′-TAD regulation in distal cells [32] is accompanied by a TAD-specific coverage of H3K27me3 marks, up to the telomeric TAD boundary where the presence of these negative marks abruptly stops (see also [54, 55]). Therefore, the TAD structure itself may dictate the extent of coverage by H3K27me3 marks, after the recruitment of PRC2 on those *Hoxd* genes switched off in these distal cells and included into this TAD.

### Heritability of polycomb-associated gene silencing

During the replication of H3K27me3-labeled DNA sequences, daughter cells inherit this histone modification from their parental cell [51, 56, 57]. Since limb development occurs mainly through a distal outgrowth, the distal cells where 5′-TAD enhancers are at work derive from proximal cells that were previously under the control of 3′-TAD enhancers. In the latter cells, the central part of the *HoxD* cluster is active and hence *Hoxd9*, *Hoxd10*, and *Hoxd11* are devoid of H3K27me3 marks, whereas *Hoxd12* and *Hoxd13*, which are located on the other side of the TAD boundary are silent and thus covered by H3K27me3 marks [33].

When these cells start to implement the 5′-TAD distal regulation, H3K27me3 marks are erased from both *Hoxd13* and *Hoxd12*, the major targets of 5′-TAD enhancers, which are transcribed at high levels. *Hoxd11* and *Hoxd9*, which are devoid of H3K27me3 marks also become transcribed in distal cells, even though their genuine function in these cells has not been unequivocally demonstrated [39]. In the absence of 3′-TAD regulation in *inv(attP-Itga6)* proximal mutant cells, the entire *HoxD* cluster is heavily covered by H3K27me3 marks since all *Hoxd* genes are silenced. When these mutant distal cells start to implement the 5′-TAD regulation, the H3K27me3 marks covering *Hoxd13* and *Hoxd12* are removed with the same kinetics as in wild-type distal cells, due to a comparable transcriptional context. However, *Hoxd11* and *Hoxd10* transcription onset is severely delayed when compared to control distal cells, as these genes were inherited in a silenced rather than active state, covered by H3K27me3 marks [33]. In this latter case, the strength of distal limb enhancers and the proximity of *Hoxd13* and *Hoxd12* likely lead to a progressive removal of PRC silencing and a weak and delayed activation of both *Hoxd11* and *Hoxd10* in distal cells. This observation illustrates both the capacity for cells to memorize their coverage in H3K27me3 marks in a physiological context and the labile aspect of polycomb silencing, which can be efficiently removed through a strong transcriptional activation.

## Conclusions

From this study, we conclude that proximal and distal limb enhancers, which are normally segregated between the two TADs flanking the *HoxD* cluster, were not dramatically affected neither in their activation nor in their specificities, when their target genes were deleted and the two TADs merged into a single chromatin interaction domain. However, in the fused TAD, the proximal regulation seems to take the lead over the distal regulation. Secondly, these results suggest a mechanism whereby the silencing of remote enhancers is accompanied by a far-*cis* spreading of polycomb group proteins after being recruited for the most part at the *HoxD* cluster itself. Finally, we conclude that active genes are more readily accessible to a subsequent enhancer regulation when compared to silenced genes, illustrating the potential importance of polycomb-associated chromatin marks in the proper timing of gene activation during development.

## Methods

### Animal experimentation and mouse strains

Genetically modified mice were kept on a (Bl6XCBA) background and crossed in heterozygosis. Distal and proximal forelimb tissues were dissected and processed from E12.5 mouse embryos. All mutant mice used in this study and their genotyping strategies have been previously described in [34, 37, 40]. Homozygous mutant embryos were obtained by crossing heterozygous mice.

### 3D modeling of Hi-C datasets

Hi-C original datasets from wild-type *HoxD*^*del(1-13)d9lac*^ and *HoxD*^*del(attP-Rel5)d9lac*^ were obtained from [34] (GEO accession: GSE101715). Three-dimensional modeling of the normalized 40 kb binned Hi-C matrices was performed by means of the model_and_analyze.py script from the TADbit v0.2.0.58 software [36] in chr2: 73800001-75760000 (wild-type coordinates mm10). We generated 500 models for optimization and 5000 for modeling, and matrix columns showing no interactions were not filtered out. We visualized the model with TADkit using the Virtual Research Environment (https://vre.multiscalegenomics.eu/home/) [58]. Region CS38-41 (wild-type coordinates in mm10, chr2:75120051-75165771) was used as a reference mark in the 3D reconstructed Hi-C model. The analysis of A/B compartments was performed using CscoreTool [59]. Briefly, the *cis* valid pairs on chr2 [34] of the wild-type and mutant Hi-C mapped on mm10 were converted to the same format as the Hi-C summary file format for HOMER. Then, CscoreTool was executed using 40 kb bins and a minDis of 1 Mb. The values of the bedgraphs with *C*-scores were inverted when they began with positive values. All bedgraphs were then loaded in the UCSC browser.

### RNA extraction, RNA-seq, and qPCR

Limb tissue was dissected, placed in RNAlater (Invitrogen), and directly frozen at − 80 °C until further processing. After genotyping, RNA was extracted from individual samples using RNAeasy Micro Kit (QIAGEN). Libraries were prepared from 100 ng of total RNA using the TruSeq Stranded mRNA protocol and sequenced on a HiSeq 2500 machine (100 bp reads, single end). Sequencing data were treated using the facilities of the Scientific IT and Application Support Center of EPFL. The gtf file used for STAR and cufflinks, based on Ensembl version 94 annotations, is available on figshare [60]. Adapters were removed using cutadapt (v1.16) and aligned onto mm10 using STAR [61] with ENCODE parameters. Normalized read counts were obtained by DESeq2 analysis, which was performed with default parameters. Genes with absolute log2 fold change above 1.5 and adjusted *p* value below 0.05 were considered as significant ([62] version 1.22.1). For *HoxD*^*del(attP-Rel5)d9lac*^ and their associated control samples, three biological replicates were used for each genotype and tissue. For plotting distal RNA-seq comparisons between wild type and *HoxD*^*del(attP-Rel5)d9lac*^ (see Additional file 2E), the 10 most differentially expressed genes in distal wild-type tissue were ranked and subsequently compared between mutant and control samples. For *HoxD*^*inv(attP-Itga6)*^ RNA-seq, only one sample was used per tissue and genotype. Track profiles show the mean of the coverage of uniquely mapped reads normalized to the number of uniquely mapped reads. They were obtained with the UCSC browser. For qPCR, purified RNA was retrotranscribed with Promega GoScript Reverse Transcriptase (Promega). Custom SYBR probes were used for quantitative real-time PCR (qPCR) in a QuantStudio 5384-well block machine. Island3 primers were forward: TTCCATCACAGGAGAGTCGTTG and reverse: AGGTGGGAACATGGACTGAAAG. All other primers were described in [39, 63]. qPCR fold inductions were considered significant when Welch’s *t* test statistical analyses showed *p* value < 0.05.

### 4C-seq experiments

The limb samples used in this study were dissected from E12.5 forelimb buds for all wild-type and mutant lines. The samples were processed as in [34]. Briefly, cellular suspensions were filtered and fixed using a 2% formaldehyde/10% FBS/PBS solution for 10 min. NlaIII (NEB) was used as the first cutter and DpnII (NEB) as the second cutter. DNA libraries were prepared using 12 to 14 independent PCR reactions with 100 ng of DNA on each. Sequencing was performed by multiplexing several independently barcoded viewpoints. 4C-seq data were analyzed using the HTSstation web interface [64]. They were normalized to the distribution of reads on a 10-Mb window, and the profiles were smoothened using a window of 11 fragments. 4C-seq data from wild-type tissue was taken from GEO (GSE101717). Data for the CS38 viewpoint were taken from GSM2713679 and for the *Hoxd4* viewpoint from GSM2713671 and GSM2713672.

### Chromatin immunoprecipitation (ChIP)

For all samples, limb tissues were dissected and directly fixed with 1% formaldehyde in PBS for 10 min at room temperature, followed by 3 min incubation with Stop Solution from the ChIP-IT High Sensitivity Kit (Active Motif). The samples were then washed 3 times with working Washing Solution (ChIP-IT, Active Motif) and then snap-frozen in liquid nitrogen and stored at − 80 °C until further processing. After genotyping, the samples were pooled according to the required cell number. The total amount of tissue used for each line was different due to the size variations of the limb buds. Limb tissues were disrupted with a polytron device, lysed in RIPA buffer or Prep Buffer (ChIP-IT, Active Motif), and sonicated in Diagenode Bioruptor Pico. All H3K27ac ChIP experiments were processed as ChIP-seqs using the reagents from ChIP-IT High Sensitivity Kit (Active Motif). IPs were performed in parallel technical duplicates with 11 to 14 μg of chromatin on each. Antibody incubation was performed overnight on a final volume of 1.5–2 ml dilution buffer (0.1% SDS, 50 mM Tris-HCl pH 8, 10 mM EDTA pH 8, and proteinase inhibitors), including 2 μl of H3K27ac antibody (Diagenode C15410196) at 4 °C on a rotating platform. Agarose beads were added for 3 to 4 h at 4 °C. Washes were performed on column, and DNA purification was carried out by phenol-chloroform extraction. The technical replicates were merged and yielded 1.5 to 2 ng of chromatin, which were used to generate DNA libraries using the TruSeq ChIP library preparation kit. The number of biological replicates used for H3K27ac ChIP experiments is shown in the figure. RING1B ChIP experiments were processed as for ChIP-seq using 4 μl of RING1B antibody (Active Motif 39664) and following the protocol described in [32]. Three and two biological replicates were processed for distal and proximal RING1B ChIP experiments, respectively.

All H3K27me3 and EZH2 ChIP were performed following the ChIPmentation protocol [65]. Around 0.1 to 0.4 million cells were used for each IP on a final volume of 800 to 1000 μl of RIPA-LS buffer (10 mM Tris-HCl pH 8, 140 mM NaCl, 1 mM EDTA pH 8, 0.1% SDS, 0.1% sodium deoxycholate, 1% Triton X-100, and proteinase inhibitors), to which 2 μl of H3K27me3 (Millipore 17-622) or EZH2 (Diagenode C15410039) antibodies were added. The samples were incubated for at least 2 h with Dynabeads Protein A (Invitrogen 10001D) rotating at 4 °C. Washes were performed as follows: two times RIPA-LS, two times RIPA-HS (10 mM Tris-HCl pH 8, 500 mM NaCl, 1 mM EDTA pH 8, 0.1% SDS, 0.1% sodium deoxycholate, 1% Triton X-100, and proteinase inhibitors), two times RIPA-LiCl (10 mM Tris-HCl pH 8, 250 mM LiCl, 1 mM EDTA pH 8, 0.5% NP-40, 0.5% sodium deoxycholate, and proteinase inhibitors), and once with 10 mM Tris-HCl pH 8. The beads were resuspended in 24 μl of tagmentation buffer (10 mM Tris pH 8, 5 mM MgCl_2_, 10% dimethylformamide) and 1 μl of Tn5 transposase (Illumina 15027865, from Nextera DNA Library Prep Kit 15028212) and transferred to PCR tubes, which were then incubated at 37 °C for 5 min in a thermocycler. The samples were then resuspended and washed twice in 1 ml of RIPA-LS and twice in 1 ml TE buffer (10 mM Tris-Hcl pH 8, 1 mM EDTA pH 8). The beads were magnetized, and DNA was eluted in ChIP elution buffer (10 mM Tris-HCl pH 8, 5 mM EDTA pH 8, 300 mM NaCl, 0.4% SDS) with 2 μl of proteinase K (20 mg/ml stock) and then incubated for 1 h at 55 °C and 6 h to overnight at 65 °C. After de-crosslinking, the supernatant was recovered and the beads were resuspended again in 19 μl ChIP elution buffer with 1 μl of proteinase K and left 1 h at 55 °C. The two supernatants were combined and purified with MinElute kit (Qiagen) in 22 μl of EB buffer. Relative quantitation was performed using SYBR Green (as in [65]) using 2 μl of DNA. Libraries were amplified according to the Cq values obtained in the previous step (12 to 14 cycles for both sets of samples), purified using Agentcourt AMPureXP beads (Beckman Coulter A63880) and eluted in 15 μl of water. DNA sequencing was performed in HiSeq 2500 or HiSeq 4000 machine as 50 bp single reads or 100 bp single reads. Only one experiment per tissue was performed for the EZH2 ChIP. The number of biological replicates used for H3K27me3 ChIP experiments is shown in the figure.

### ChIP analysis

Analyses were performed using the facilities of the Scientific IT and Application Support Center of EPFL. Sequences were filtered, and adapters were removed using cutadapt (v1.16) [66] with parameters -m 15 -q 30 -a CTGTCTCTTATACACATCTCCGAGCCCACGAGAC for ChIPmentation and -a GATCGGAAGAGCACACGTCTGAACTCCAGTCAC for ChIP-seq. Reads were mapped on mm10 using bowtie2 (v2.3.4.1) using default parameters [67]. Only reads with a mapping quality above 30 were kept. A profile was obtained with macs2 [68] (version 2.1.1.20160309 option –extsize 300). Bedgraphs were normalized to their number of million tags used in the profile, and replicates were merged using the tool unionbedg (bedtools v2.27) [69]. The profiles were loaded in the UCSC browser with "mean" as windowing function. The difference profiles were calculated using unionbedg. In order to quantify the gain or loss of chromatin marks in the 3′-TAD, and in the CS38-41 region, the number of reads falling into their respective intervals (chr2:74781516-75605516 for the 3′-TAD and chr2:75120051-75165771 for the CS38-41 region) were assessed after duplicate removal by picard (http://broadinstitute.github.io/picard/ version 2.18.14) [70] using the multiBamSummary function from deeptools [71]. For the 5′-TAD, two different ranges were taken into account depending on whether the full regulatory domain was intact in the mutant configuration: chr2:73914154-74636454 when comparing wild type, *HoxD*^*inv(attP-Itga6)*^, and *HoxD*^*inv(Nsi-Itga6)*^ alone (Fig. 5, Additional file 3) and chr2:73914154-74422050 whenever *HoxD*^*del(attP-Rel5)d9lac*^ was included in the comparisons (Fig. 3, Additional file 4). Also, for the 5′-TAD, the reads falling into the region of the artifactual peak, which is due to a PCR contamination (chr2:74207282-74208158), were excluded in all datasets. The counts were normalized to the number of reads in the input bam file, and the significance (Wilcoxon rank-sum test) was assessed by the function wilcox.test in R (https://www.R-project.org).

### Whole-mount in situ hybridization and beta-galactosidase staining

Island3, *Hoxd4*, *Hoxd8*, *Hoxd9*, *Hoxd11*, *Hoxd13*, and *Evx2* WISH were performed following the protocol described in [72]. The DNA fragment for Island3 probe was amplified from purified genomic DNA using primers GCAGGAATGACAGACAGGCA (Fw) and ACAGAGGTGGGAACATGGAC (Rv) and cloned into pGEM-T easy vector (Promega A1360). Beta-galactosidase staining was performed as in [34]. *Hoxd4*, *Hoxd8*, *Hoxd9*, *Hoxd11*, *Hoxd13*, and *Evx2* probes were as in [73].

## Additional files

Additional file 1: 3D-representation of the *HoxD* locus in *del(1-13)d9lac*-mutant limb buds. (*A*) Hi-C map showing the presence of both TADs on either side of the *HoxD* locus in *del(1-13)d9lac* proximal limb cells and its associated genes (gray boxes) and regulatory regions (black and red boxes) (*B*, *C*) TADkit-derived 3D representation of Hi-C datasets [34] obtained for distal (B) and proximal (C) limb cells processed from *del(1-13)d9lac*-mutant mice. The CS38-41 region is shown as a red disk in the 3D models to be used as a reference point. In this deletion allele, both TADs are still visible unlike in the larger *del(attP-Rel5)d9lac* deletion shown in Fig. 1. (*D*) A/B compartment distribution along chromosome 2. *C*-scores were calculated from *Wt* and *del(attP-Rel5)d9lac* E12.5 distal and proximal limb Hi-C data. Compartment A is represented as positive values (red) and compartment B as negative values (blue). Gene density is shown in the bottom panel and the *HoxD* locus is indicated as a blue bar. (PDF 814 kb)

Additional file 2: Expression analysis around the *HoxD* associated transcripts. (*A*) WISH of *lacZ* mRNA in *del(attP-Rel5)d9lac* and *del(1–13)d9lac* E11.5 forelimbs (center) and whole embryos (left). The proximal domain is shown by an arrowhead. *Hoxd10* and *Hoxd11* WISH of E11.5 wild-type forelimbs are shown on the right for comparison of their expression domains. (*B*) WISH of Island3 eRNAs in *Wt* and *del(attP-Rel5)d9lac* E12.5 embryos where the antisense probe was used (left) showing the specificity on the digital region. On the right, a probe control shows the lack of staining with the Island3 sense probe. (*C*, *D*) Volcano plots of all genes analyzed by RNA-seq in proximal (C) and distal (D) limb tissues comparing *del(attP-Rel5)d9lac* expression values to the control. All the genes located inside or in the vicinity of the 5′-TAD and the 3′-TAD are marked in red. Blue dots represent differentially expressed genes (absolute log2 fold change above 1.5 and adjusted *p* value below 0.05) that are located outside these regions. *Hoxd9* and *Gm28793* (a short antisense mRNA) are significantly expressed due to their presence inside the *Hoxd9/LacZ* transgene. (*E*). RNA-seq-normalized read counts of wild-type versus *del(attP-Rel5)d9lac* distal limb tissue. The ten genes were selected according to the highest fold induction values obtained when wild-type distal and proximal tissues were compared. Statistical significance was assessed as adjusted *p* values of the DESeq2 analysis. (PDF 14200 kb)

Additional file 3: H3K27me3 distribution after disconnecting the *HoxD* from its 3′-TAD regulatory landscape. H3K27me3 ChIP profiles from proximal limb bud cells derived from either wild type, *inv(attP-Itga6)*, or *inv(Nsi-Itga6)* specimens. *n* indicates the number of replicates for each track. Below each mutant dataset, a comparison of mutant versus control is shown. A red asterisk in the control track indicates an artifactual signal at the position of island-3. (PDF 537 kb)

Additional file 4: (*A*) H3K27me3 coverage outside the *HoxD* locus in distal limb bud cells from either control (*Wt*) or *del(attP-Rel5)d9lac* specimens. A corresponding Hi-C map is shown on top spanning ca. 10 Mb and centered around the *HoxD* cluster (blue box), with the related TAD structures (chr2:69600001-79440000). The flanking 5′ and 3′-TAD TADs are indicated. H3K27me3 ChIP profiles in control cells show a global coverage outside the *HoxD* cluster precisely restricted to the 3′-TAD. In *del(attP-Rel5)d9lac*-mutant distal limb cells, the enrichment is much weaker. Below is the difference in the ChIP datasets comparing mutant versus control signals. The red asterisks point to an artifactual signal. (*B*) Quantification of H3K27me3 ChIP signal of wild type, *del(attP-Rel5)d9lac*, *inv(attP-Itga6)*, and *inv(Nsi-Itga6)* in distal (left) or proximal (right) forelimb cells. The plotted values are computed from the regions depicted in (A) as dashed boxes. (PDF 2145 kb)

Additional file 5: H3K27me3 signal over the *HoxD* cluster in the absence of the 5′-TAD. H3K27me3 ChIP profiles in distal (top two tracks) and proximal (bottom two tracks) limb bud cells, either in control (*Wt*) or in *inv(Nsi-Itga6)* mutant specimen. Below are shown the difference profiles. The increase of signal in *Hoxd11* represents reads coming from the *Hoxd11lac* transgene included in the inversion allele (represented by a shaded gray box). (PDF 429 kb)

## Data Availability

The datasets generated and analyzed for this study are available in the GEO repository under accession number GSE129427 (https://www.ncbi.nlm.nih.gov/geo/query/acc.cgi?acc=GSE129427) [74]. Publicly available data used in this paper can be found in GEO under the accession numbers GSE101715 (Hi-C) [34] and GSE101713 (4C-seq) [34]. The gtf file used for STAR and cufflinks, based on Ensembl version 94 annotations, is available on figshare [60].

## Abbreviations

3′-TAD: Telomeric TAD
5′-TAD: Centromeric TAD
ChIP: Chromatin immunoprecipitation
eRNA: Enhancer RNA
lncRNA: Long non-coding RNA
PRC: Polycomb repressive complex
TAD: Topologically associating domain
WISH: Whole-mount in situ hybridization
