# Supplementary figures and images for: Impact of genome architecture on the functional activation and repression of Hox regulatory landscapes
Source: BMC Biol. 2019 Jul 12;17:55. doi: 10.1186/s12915-019-0677-x (PMC6626364; doi:10.1186/s12915-019-0677-x)

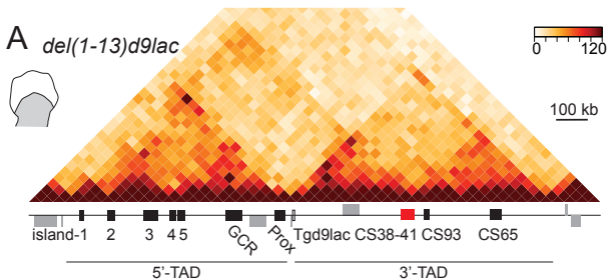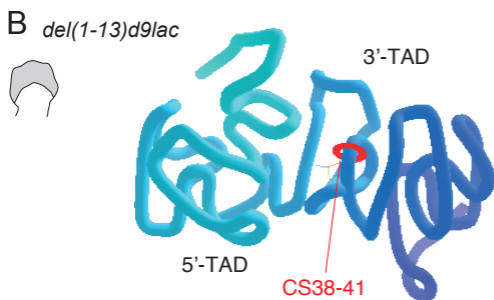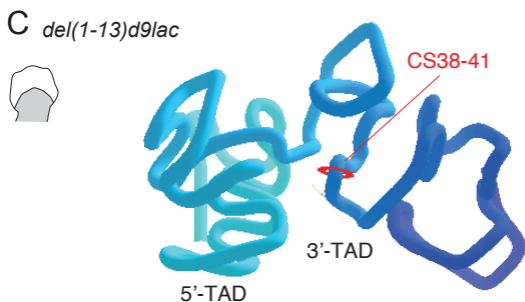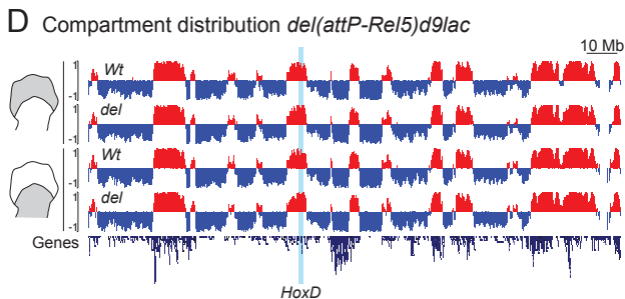

Supplement: Supplementary file 1 — 3D-representation of the HoxD locus in del(1-13)d9lac-mutant limb buds. (A) Hi-C map showing the presence of both TADs on either side of the HoxD locus in del(1-13)d9lac proximal limb cells and its associated genes (gray boxes) and regulatory regions (black and red boxes) (B, C) TADkit-derived 3D representation of Hi-C datasets [34] obtained for distal (B) and proximal (C) limb cells processed from del(1-13)d9lac-mutant mice. The CS38-41 region is shown as a red disk in the 3D models to be used as a reference point. In this deletion allele, both TADs are still visible unlike in the larger del(attP-Rel5)d9lac deletion shown in Fig. 1. (D) A/B compartment distribution along chromosome 2. C-scores were calculated from Wt and del(attP-Rel5)d9lac E12.5 distal and proximal limb Hi-C data. Compartment A is represented as positive values (red) and compartment B as negative values (blue). Gene density is shown in the bottom panel and the HoxD locus is indicated as a blue bar. (PDF 814 kb) [file 12915_2019_677_MOESM1_ESM.pdf]

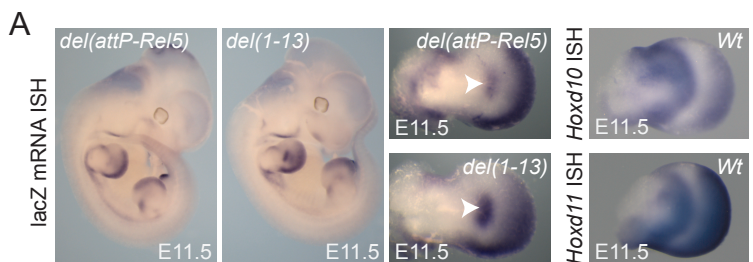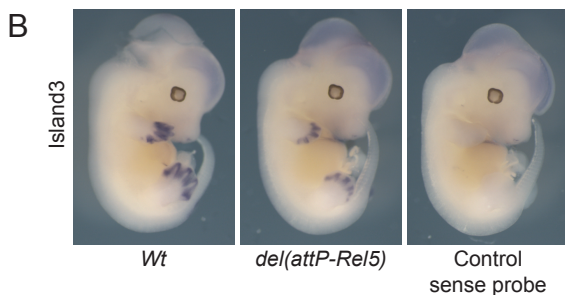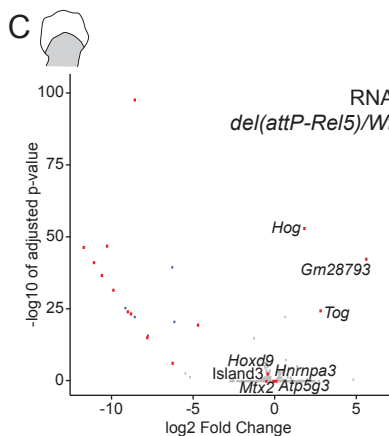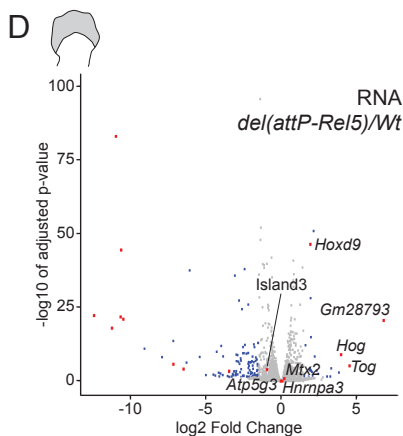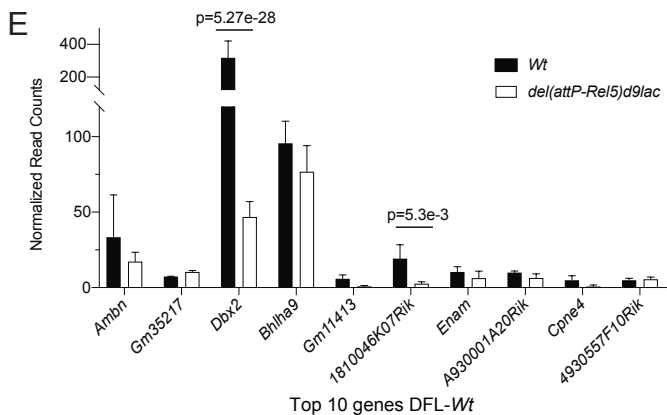

Supplement: Supplementary file 2 — Expression analysis around the HoxD associated transcripts. (A) WISH of lacZ mRNA in del(attP-Rel5)d9lac and del(1–13)d9lac E11.5 forelimbs (center) and whole embryos (left). The proximal domain is shown by an arrowhead. Hoxd10 and Hoxd11 WISH of E11.5 wild-type forelimbs are shown on the right for comparison of their expression domains. (B) WISH of Island3 eRNAs in Wt and del(attP-Rel5)d9lac E12.5 embryos where the antisense probe was used (left) showing the specificity on the digital region. On the right, a probe control shows the lack of staining with the Island3 sense probe. (C, D) Volcano plots of all genes analyzed by RNA-seq in proximal (C) and distal (D) limb tissues comparing del(attP-Rel5)d9lac expression values to the control. All the genes located inside or in the vicinity of the 5′-TAD and the 3′-TAD are marked in red. Blue dots represent differentially expressed genes (absolute log2 fold change above 1.5 and adjusted p value below 0.05) that are located outside these regions. Hoxd9 and Gm28793 (a short antisense mRNA) are significantly expressed due to their presence inside the Hoxd9/LacZ transgene. (E). RNA-seq-normalized read counts of wild-type versus del(attP-Rel5)d9lac distal limb tissue. The ten genes were selected according to the highest fold induction values obtained when wild-type distal and proximal tissues were compared. Statistical significance was assessed as adjusted p values of the DESeq2 analysis. (PDF 14200 kb) [file 12915_2019_677_MOESM2_ESM.pdf]

# Rodríguez-Carballo et al. Additional File 3

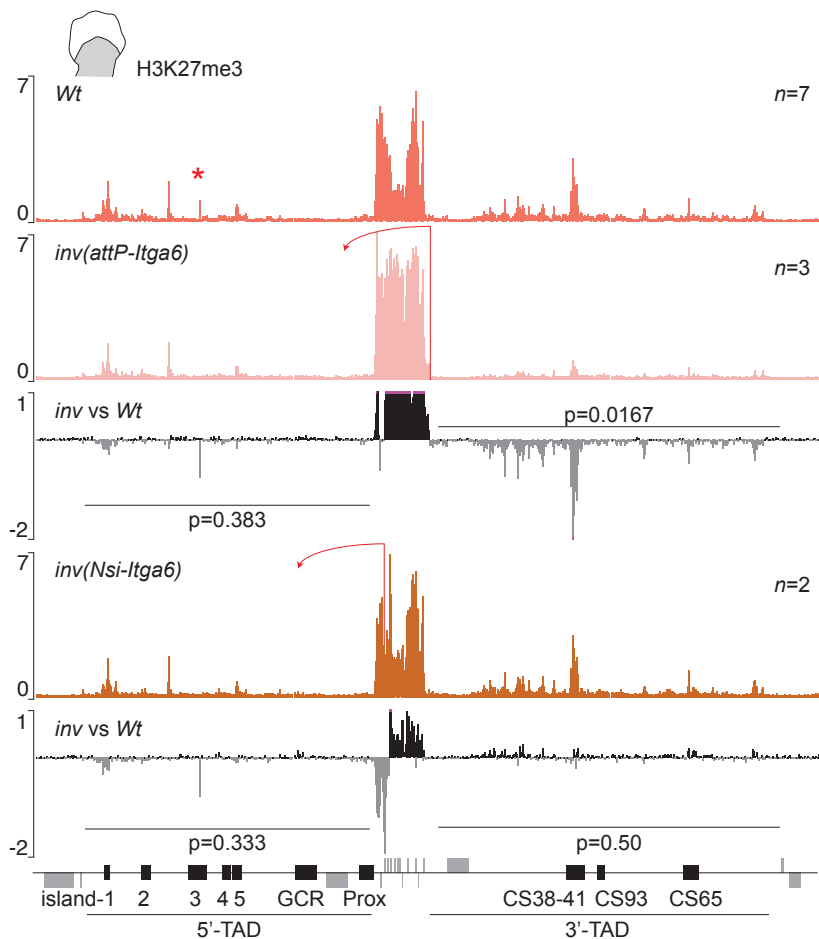

Supplement: Supplementary file 3 — H3K27me3 distribution after disconnecting the HoxD from its 3′-TAD regulatory landscape. H3K27me3 ChIP profiles from proximal limb bud cells derived from either wild type, inv(attP-Itga6), or inv(Nsi-Itga6) specimens. n indicates the number of replicates for each track. Below each mutant dataset, a comparison of mutant versus control is shown. A red asterisk in the control track indicates an artifactual signal at the position of island-3. (PDF 537 kb) [file 12915_2019_677_MOESM3_ESM.pdf]

A

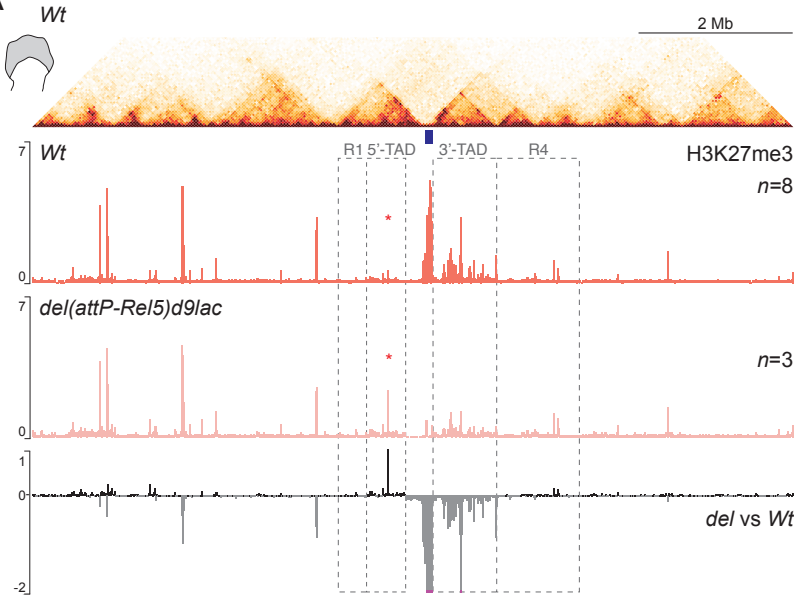

B

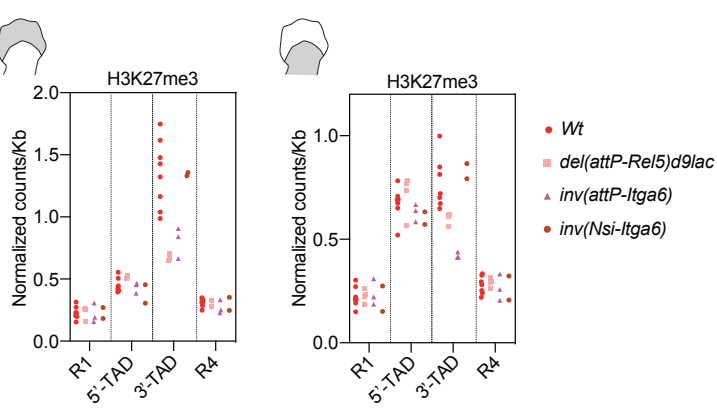

Supplement: Supplementary file 4 — (A) H3K27me3 coverage outside the HoxD locus in distal limb bud cells from either control (Wt) or del(attP-Rel5)d9lac specimens. A corresponding Hi-C map is shown on top spanning ca. 10 Mb and centered around the HoxD cluster (blue box), with the related TAD structures (chr2:69600001-79440000). The flanking 5′ and 3′-TAD TADs are indicated. H3K27me3 ChIP profiles in control cells show a global coverage outside the HoxD cluster precisely restricted to the 3′-TAD. In del(attP-Rel5)d9lac-mutant distal limb cells, the enrichment is much weaker. Below is the difference in the ChIP datasets comparing mutant versus control signals. The red asterisks point to an artifactual signal. (B) Quantification of H3K27me3 ChIP signal of wild type, del(attP-Rel5)d9lac, inv(attP-Itga6), and inv(Nsi-Itga6) in distal (left) or proximal (right) forelimb cells. The plotted values are computed from the regions depicted in (A) as dashed boxes. (PDF 2145 kb) [file 12915_2019_677_MOESM4_ESM.pdf]

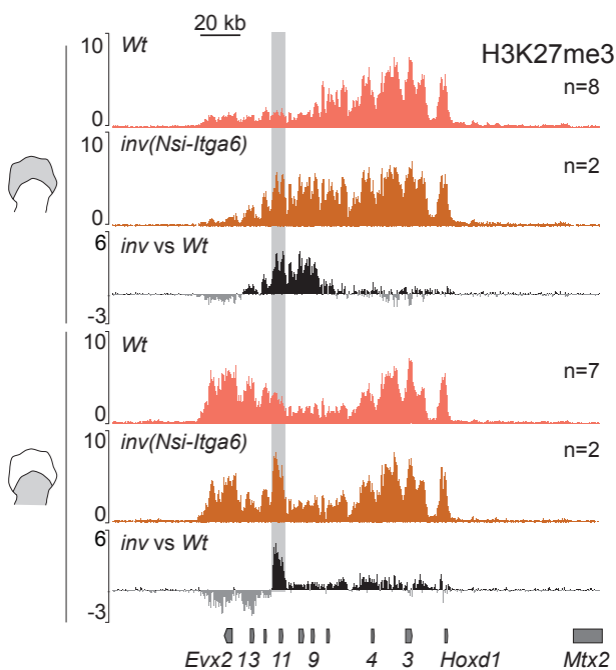

Supplement: Supplementary file 5 — H3K27me3 signal over the HoxD cluster in the absence of the 5′-TAD. H3K27me3 ChIP profiles in distal (top two tracks) and proximal (bottom two tracks) limb bud cells, either in control (Wt) or in inv(Nsi-Itga6) mutant specimen. Below are shown the difference profiles. The increase of signal in Hoxd11 represents reads coming from the Hoxd11lac transgene included in the inversion allele (represented by a shaded gray box). (PDF 429 kb) [file 12915_2019_677_MOESM5_ESM.pdf]
